# Smartphone-based gait analysis in the assessment of fatigue and fatigability in people with multiple sclerosis: a supervised cohort study

**DOI:** 10.1007/s00415-025-12906-7

**Published:** 2025-02-22

**Authors:** Carolin Schönherr, Julian Ziegler, Ton Zentek, Asarnush Rashid, Sebastian Strauss, Alexander Tallner, Matthias Grothe

**Affiliations:** 1 Department of Neurology, University Medicine Greifswald, Greifswald, Germany; 2 Innovation Management, Zentrum Für Telemedizin, Bad Kissingen, Germany

**Keywords:** Multiple sclerosis, 6MWT, Gait analysis, Fatigue, Smartphone

## Abstract

**Background:**

Gait impairments and fatigue are the most common and disabling symptoms in people with multiple sclerosis (PwMS). Objective 6-min walk test (6MWT) gait testing can be improved through body-worn accelerometers, but its association to subjective fatigue and objective fatigability is contradictory. This study aims to validate an algorithm using smartphone sensor data for spatial–temporal gait parameters in PwMS and healthy controls, and evaluate its accuracy in detecting fatigability, and quantify its association with fatigue in PwMS.

**Methods:**

We recruited PwMS with mild to moderate disability (EDSS 0.0–6.5) and healthy controls in a supervised, lab-based cohort study. All participants performed the 6MWT while wearing a smartphone at the hip, which collected acceleration data of step count, cadence and walking speed. Algorithm validation included the mean absolute percentage error (MAPE) and Bland–Altman analysis. Fatigability and fatigue were measured in PwMS, with fatigability defined as a 10% decline in gait performance, and fatigue using the fatigue scale for motor and cognitive functions (FSMC). Further, correlations between gait parameters and FSMC were assessed.

**Results:**

A total of 38 PwMS and 24 healthy controls were included. The algorithm demonstrated high validity for step count (MAPE < 3%) and cadence (MAPE < 10%). Gait analyses revealed fatigability in between 2.6 and 15.8% of PwMS, with large differences between the gait parameter assessed. Significant correlations were found especially between FSMC motor fatigue scores and step count (*r* = − 0.50), cadence (*r* = 0.51) and walking speed (*r* = 0.50).

**Conclusion:**

Smartphone-based gait analysis provides an accessible and valid method for detecting steps and cadence. There are major differences in the assessment of fatigability, but an allover association to subjective motor fatigue.

## Introduction

Gait impairments and fatigue are among the most prevalent and debilitating symptoms in people with multiple sclerosis (PwMS), significantly affecting their mobility and overall quality of life (QoL) [1–3].

The Expanded Disability Status Scale (EDSS) [4] is a widely utilized instrument in clinical practice, with gait and mobility as substantial parts of this evaluation tool. The EDSS is highly standardized, but it comes with limitations concerning its reliability and preciseness [5], especially compared to objective gait analysis. Therefore, the 6-min-walk test (6MWT) can be performed additionally to assess mobility and endurance [6]. The 6MWT is frequently used in clinical routine, and can also quantify endurance in a standardized way. Research on gait impairments in neurological disorders increased within the last years due to technological developments [7]. Body-worn accelerometers enables continuous assessment of mobility in clinical research [8], and wearables are advantageous in detecting changes in mobility, even in early stages of MS [9]. There is a large heterogeneity in the literature with regard to the physical position of the accelerometers, the investigated domain and the laboratory or real-life environment [8]. For gait analysis, several wearable devices have been developed and studied, highlighting the usage of body-worn accelerometers for the assessment of gait disturbances in PwMS [10, 11]. However, most of these devices are very expensive, which limits its wider all-day application. Another approach focusses on smartphones and applications, as they are easily accessible, allow precise measurements of different gait parameters at low cost, and can, therefore, pave the way for an independent performance of the 6MWT even in a home-based setting.

Fatigue is a highly prevalent and burdensome symptom in PwMS [2, 12], defined as a ‘decrease in physical and/or mental performance that results from changes in central, psychological, and/or peripheral factors’ [13]. Two different domains can be differentiated, the subjective perception of fatigue and the more objective fatigability, which refers to a decline of performance over time [14]. The literature so far only reveals limited association between fatigue and fatigability, which is due to a high heterogeneity in the assessment of subjective fatigue and objective fatigability [15]. For the subjective perception of fatigue, different valid questionnaires and scores already exist and can be used for diagnostics [16]. The Fatigue Score for Motor and Cognitive Function (FSMC) is an internationally validated and widely used questionnaire trying to discriminate between subjective motor and cognitive fatigue especially in MS [17].

The 6MWT is commonly used as an objective measure to assess changes in walking performance and detect signs of fatigability in PwMS. This is often demonstrated by comparing gait parameter declines between the first and sixth minutes of the test [18–20]. Although the 6MWT is frequently integrated into clinical practice, only a limited number of studies have employed wearable devices to monitor gait during the test [21–23]. To our knowledge, smartphone sensors have never been investigated in combining the objective assessment of gait and the subjective domain of fatigue.

Therefore, the aims of our study are:

1. To validate an algorithm using smartphone sensor data to identify steps, cadence and walking speed in mildly and moderately affected PwMS and healthy controls during a 6 MWT.
2. To assess sensitivity and specificity of the algorithm with regard to detecting a predetermined fatigability criterion (performance in first vs sixth minute of the test) [18] for steps, cadence and walking speed as the most commonly used gait parameters [10, 11] and
3. To quantify the association between smartphone-based 6MWT parameters and subjective measures of fatigue.

## Methods

### Participants and recruitment

We recruited PwMS and healthy controls (university students or employees) at the Department of Neurology at the University medicine Greifswald, Germany, between 01/2022 and 06/2023. Eligible participants provided informed, written consent prior to participation. This study was approved by the local ethics committee at the Greifswald University medicine (BB136/21).

PwMS were recruited with the following inclusion and exclusion criteria.

Inclusion criteria:

- Diagnosis of multiple sclerosis according to the McDonald criteria [24].
- Age 18–65.
- Mild to moderate disability, assessed with the expanded disability status scale (EDSS)[4] 0–6.5; i.e., the ability to walk at least 200 m with or without walking aid.
- Written informed consent.

Exclusion criteria:

- Severe cardio-vascular diseases, cognitive impairment or comorbidity leading to reduced mobility.
- Relapse or administration to glucocorticoids within the last 3 months.
- Usage of orthoses.
- Inability to use a smartphone.

We divided the PwMS into mildly (EDSS < 4) and moderately affected (EDSS $\ge$ 4) group [25] for further analyses.

For healthy participants, inclusion criteria were age 18–65, and written informed consent. Exclusion criteria were the same as for PwMS.

### Assessments

#### Walking test procedures

**Fig. 1:**
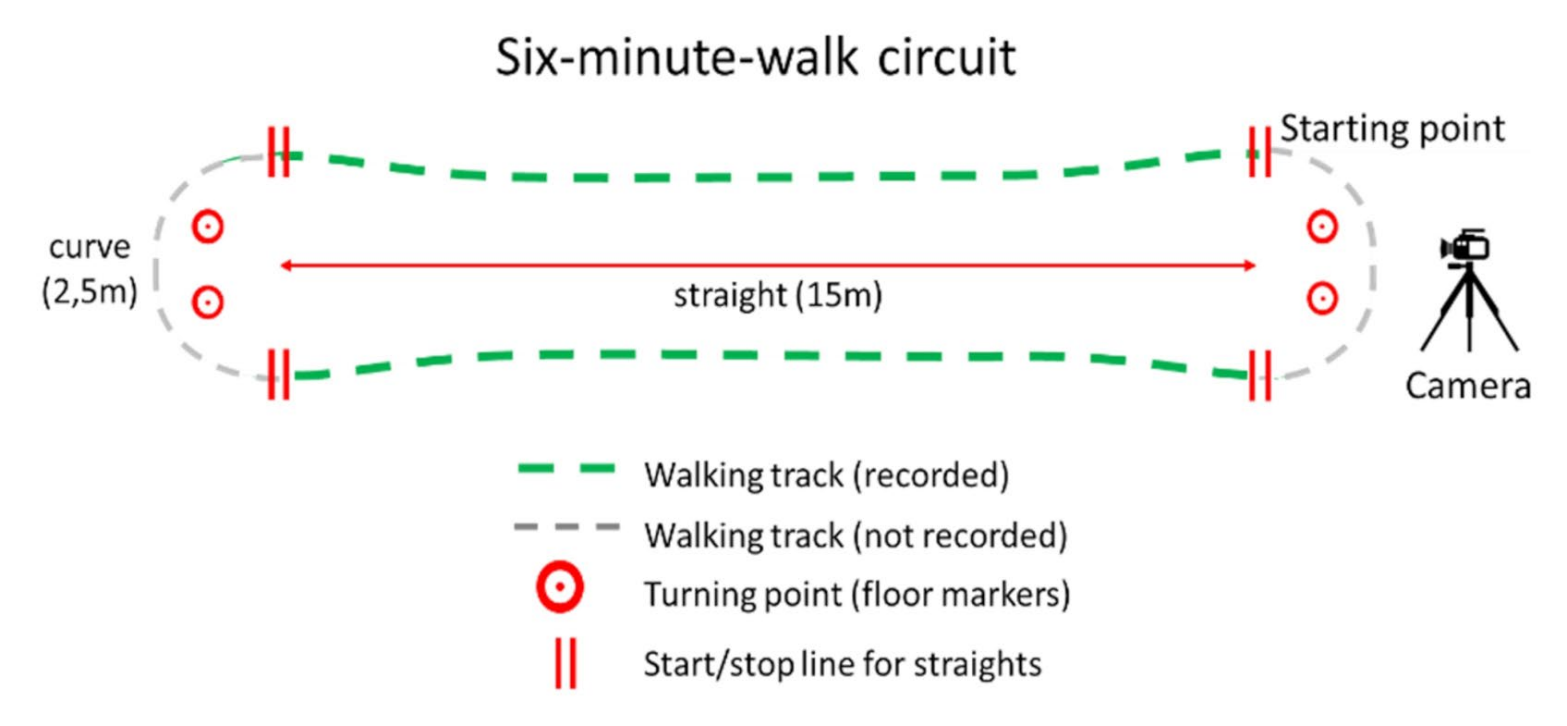
Test setup for the 6-min-walk circuit

We chose the 6-min walk test (6MWT) [26] as one of the most often used clinical walking tests in persons with MS [27] that is suitable for our sample (EDSS 0–6.5). During the 6MWT, participants are advised to walk, not run, as far as they safely can during 6 min. The 6MWT was performed on a circuit that was setup within the University medicine Greifswald. Participants walked within a circuit on two straight segments of 15 m, connected by two curved segments of 2.5 m (Fig. 1). As recommended for the validation of step recognition by smartphones [28], a video camera was placed behind the starting point to record each test. Subjects were only video-recorded beneath the chest area to ensure privacy.

A non-blinded assessor walked shortly behind the participant to ensure safety, if participants should stumble or fall. The assessor indicated start and end time for each straight walking segment by stomping a foot onto the floor when participants swing a foot over the start line, respectively, the end line. One step was counted for each foot contact on the floor on a straight segment. Measurements were performed and documented according to a data collection protocol.

#### Sensor data acquisition and calculation of parameters

A smartphone (Nokia 8—Nokia Corporation, Espoo, Finland) was worn on a belt around the hip in a central position. It was placed in vertical orientation within a firm case. Before and after each test, participants were asked to stand still for about 10 s, to allow the assessor to start or stop measurement on the smartphone.

Raw acceleration data from smartphone sensors were collected with the smartphone application Phyphox, developed by the RWTH Aachen University (RWTH Aachen University, Aachen, Germany) [29]. At the end of the 6MWT, Phyphox raw acceleration signals were saved as a.CSV file for further analysis. Sensor data was only analyzed for the straights, not the curves.

The smartphone raw acceleration signals were analyzed with a step-detection algorithm using zero-crossing detection developed by the Zentrum für Telemedizin Bad Kissingen (ZTM, Zentrum für Telemedizin Bad Kissingen, Bad Kissingen, Germany).

The sensor data analysis involved pre-processing using a bidirectional bandpass filter and signal smoothing techniques. We employed a zero-crossing algorithm to identify individual steps, followed by filtering the results with a threshold value to eliminate isolated steps. Cadence was subsequently calculated based on the temporal data. This algorithm had already demonstrated high sensitivity (99.95%) during treadmill and overground walking [25].

We identified the vertical axis and analyzed speed in overlapping sliding windows of approximately three seconds to extract relevant features. This facilitated the calculation of feature signal energy, which was then processed using regression analysis. In addition, we used signal energy to determine the trained support vector regression model (SVR). Ultimately, these sliding windows were integrated to compute the overall speed. Acceleration signals were processed using Matlab (The MathWorks, Inc., Natick, MA, USA).

Based on smartphone data, we calculated the following variables for each straight segment: step count, step cadence (extrapolated as steps per minute), and walking speed. Those variables were then averaged for all straight segments.

To investigate fatigability, as decline in performance over time, the three gait parameters were analyzed in 6 segments, each representing one minute of the 6MWT. We analyzed performance in the first minute compared to the last minute and calculated percentage of change. In adaption to the distance walked index (DWI), that compares the distance of the first to last minute [18, 20], we considered a decline of 10% in performance as fatigability.

We compared the calculated steps with reference values that were obtained from direct observation using the video recordings. Steps were manually counted, and the time needed to complete a straight segment was calculated using the stomping signals from the assessor. Walking speed was calculated from segment duration and distance.

#### Clinical assessment

We assessed routine clinical data from all PwMS including age, gender, height, body weight, disease duration, type of MS and EDSS Score.

Self-reported walking ability was assessed with the MSWS-12/D questionnaire [30], a German adaptation of the Multiple Sclerosis walking Scale-12 [31]. Each of the 12 items is answered on a 5-point Likert scale with scores from 1 to 5. The scores for all items are added up to a total score ranging from 12 to 60, with higher scores representing higher levels of walking impairment. The total score is transformed into the walk12-score ranging from 0 to 100 ((total score − 12)/48 × 100 = walk12-score).

Fatigue was assessed with the Fatigue Scale for Motor and Cognitive Functions (FSMC) [17]. The FSMC is a self-reporting 20-item questionnaire differentiating between motor and cognitive functions and determining the severity of fatigue (mild, moderate, severe). Items are rated on a 5-point Likert scale with scores from 1 to 5, so that a total maximum score of 100 can be reached, 50 for each 10-item function category. A total score of 43 and higher is classified as fatigue, distinguishing between mild ($\ge 43$), moderate ($\ge 53$) and severe fatigue ($\ge 63$). In the subscales, cognitive and motor fatigue cutoff values are at 22 points.

### Statistics

#### Statistical analysis

Z-values were calculated for each variable to identify possible outliers.

To evaluate the validity of sensor-based gait analysis, we used the mean absolute percentage error (MAPE) and the Bland–Altman limits of agreement as suitable methods [28]. Differences between calculated and observed variables are expressed as mean differences and standard deviations, and as the MAPE for step count, cadence and walking speed. For a visual comparison and agreement of sensor-based data and observed reference values, Bland–Altman analyses with calculation of limits of agreement (LOA) were used.

Correlations between subjective fatigue, assessed with the FSMC, and steps, cadence and walking speed of the 6MWT as well es differences of the first to sixth minute were analyzed using Pearson’s r. Bonferroni correction was applied to adjust p values for multiple comparisons. Statistical analyses were performed using IBM SPSS Statistics 25 (IBM, Armonk, NY, USA).

#### Validity criteria

In accordance with previous studies and standards [25], a MAPE < 3% was considered acceptable for step count and cadence. To our knowledge, there is no accepted standard for MAPE concerning walking speed. We assumed that a MAPE of < 10% is still acceptable.

#### Fatigability criteria

Adapted to the distance walked index (DWI) [20], a decline of 10% in performance from the first minute to the last minute of the 6MWT was considered as task-related fatigability.

## Results

### Sample characteristics

A total of 43 PwMS, with an EDSS ranging from 0.0 to 6.5, and 24 healthy controls participated in this study. 5 participants of the PwMS group had to be excluded from analysis, due to various reasons: three were unable to finish the 6MWT without pausing, one had erroneous data recording, and one exhibited extremely unstable walking, identified statistically as an outlier. All further analyses were carried out for the remaining 38 PwMS.

Table 1 reports data for all observed demographics and clinical characteristics of included participants. On average, healthy controls walked 128.1 m further with 60.82 more steps and higher cadence and walking speed compared to PwMS. 84.2% of PwMS reported subjective fatigue in the FSMC, 89.5% in the motor subscale for fatigue. Moderately affected PwMS reached higher scores in all subjective measures (FSMC, MSWS-12) than mildly affected persons.

**Table 1:** Demographics and clinical characteristics of the participants reporting observed values

	PwMS total Mean (SD)	PwMS mildly affected Mean (SD)	PwMS moderately affected Mean (SD)	Healthy controls Mean (SD)
N	38	23	15	24
Disease Course (RRMS/SPMS/PPMS)	31/2/5	22/0/1	9/2/4	
Sex Female Male	27 11	16 7	11 4	12 12
Age	41.6 (11.47)	36.17 (10.29)	49.93 (7.66)	27.7 (7.59)
Height in cm	171.8 (8.46)	172.52 (7.69)	170.67 (9.70)	175.71 (10.58)
Weight in kg	75.47 (16.85)	75.10 (14.93)	76.00 (19.97)	73.21 (15.49)
EDSS*	2.93 (1.70)	1.74 (0.78)	4.77 (0.86)	-
6MWT distance (straights)	344.39 (89.82)	399.34 (54.15)	260.15 (64.40)	472.49 (47.75)
6MWT total steps	513.26 (61.80)	546.70 (40.62)	462.00 (53.38)	574.08 (32.54)
6MWT steps per straight segment	22,77 (4.04)	20.33 (1.66)	26.51 (3.74)	18.14 (1.64)
6MWT cadence	107.07 (11.74)	125.59 (7.66)	97.90 (11.08)	119.00 (6.38)
6MWT walking speed	1.20 (0.30)	1.38 (0.18)	0.92 (0.22)	1.63 (0.16)
FSMC total	61.89 (19.79)	56.09 (18.49)	70.80 (18.93)	-
MSWS-12	28.26 (13.41)	19.65 (6.40)	41.47 (10.11)	-

^*^EDSS range: 0.0–6.5 = PwMS Total; 0.0–3.0 = mildly affected PwMS; 4.0–6.5 = moderately affected PwMS

### Sensor-acquired 6MWT parameters and validation

Mean 6MWT parameters and their standard deviation determined by the smartphone algorithm are reported in Table 2. The mean absolute percentage error (MAPE) for validation fulfilled the criteria for acceptance (MAPE < 3%) for step count and cadence. MAPE for walking speed of 4.744 in healthy controls and 8.137 in mildly affected PwMS were below the assumed limit of acceptance 10%, whereas they exceeded in the total and moderately affected PwMS groups.

**Table 2:** Mean 6MWT sensor-acquired gait parameters with standard deviations (SD) and mean absolute percentage error (MAPE)

	Mean (SD)				MAPE in %			
	Total PwMS	Mildly affected	Moderately affected	Healthy controls	Total PwMS	Mildly affected	Moderately affected	Healthy controls
6MWT total steps	510.53 (62.80)	545.13 (39.83)	457.47 (54.27)	569.63 (30.69)	0.917	0.674	1.289	0.949
6MWT steps per straight segment	22.62 (3.91)	20.28 (1.71)	26.22 (3.78)	17.99 (1.62)	0.918	0.675	1.292	0.947
6MWT cadence	107.91 (11.95)	113.90 (7.72)	98.71 (11.58)	119.07 (6.06)	1.031	0.833	1.334	0.542
6MWT walking speed in m/s	1.31 (0.28)	1.39 (0.22)	1.15 (0.31)	1.61 (0.10)	14.484*	8.137	26.251*	4.744

*MAPE above 10% is considered as not accepted

**Fig. 2:**
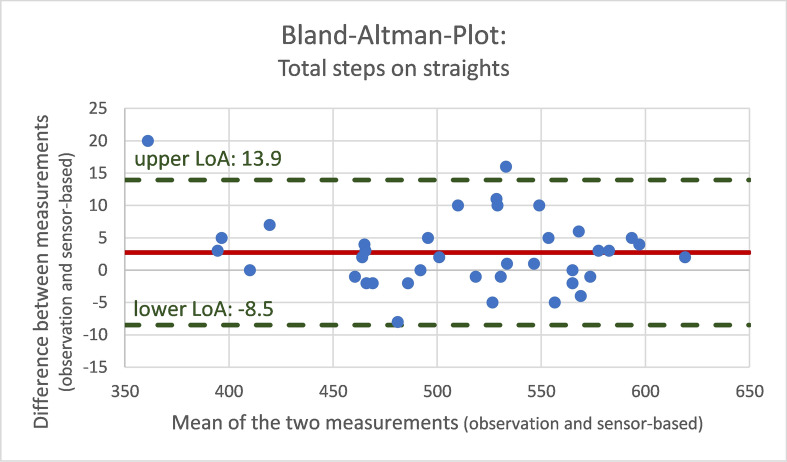
Bland–Altman plot for total steps on straights comparing the different measurements (video-documented observation and sensor-based assessment)

**Fig. 3:**
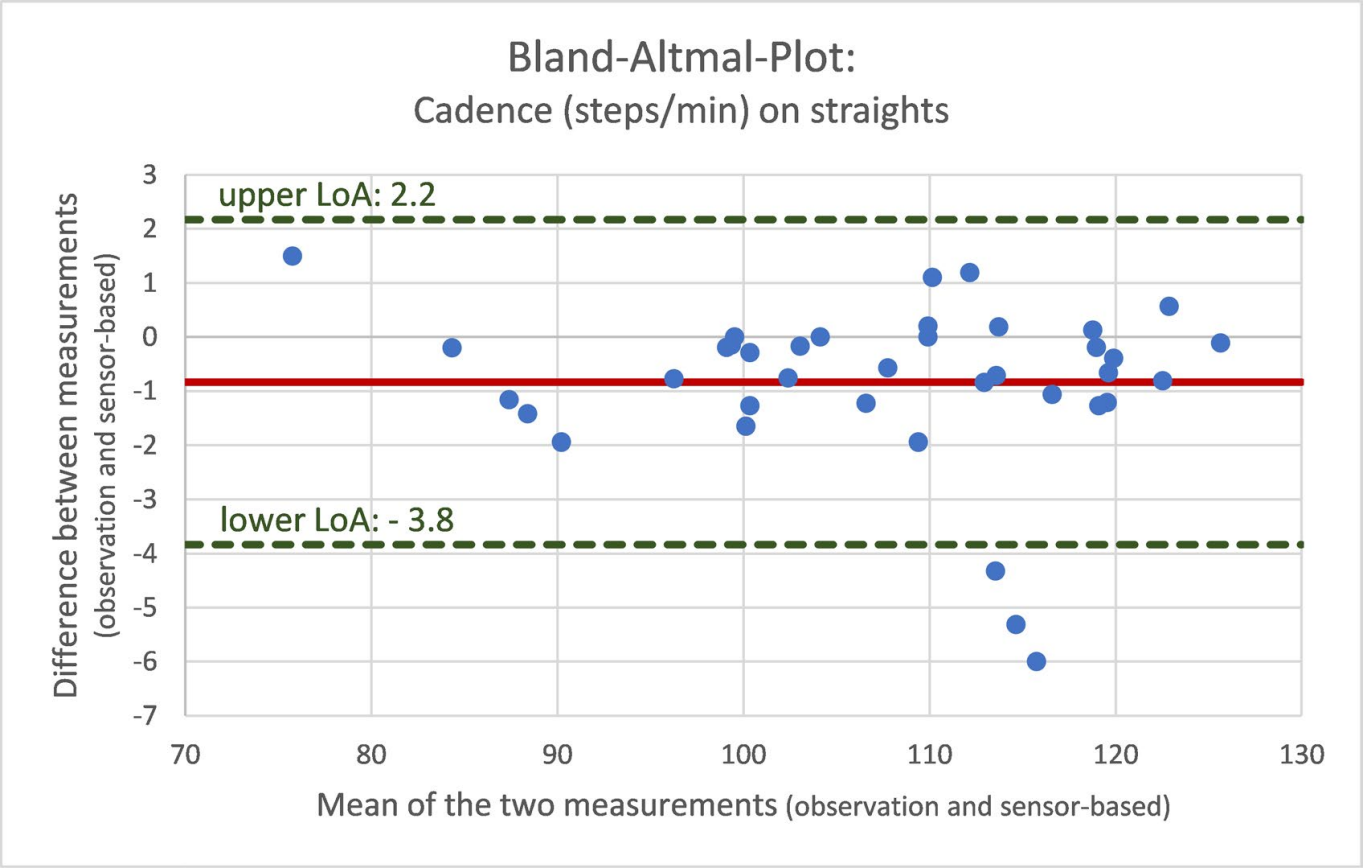
Bland–Altman plot for cadence on straights comparing the different measurements (video-documented observation and sensor-based assessment)

**Fig. 4:**
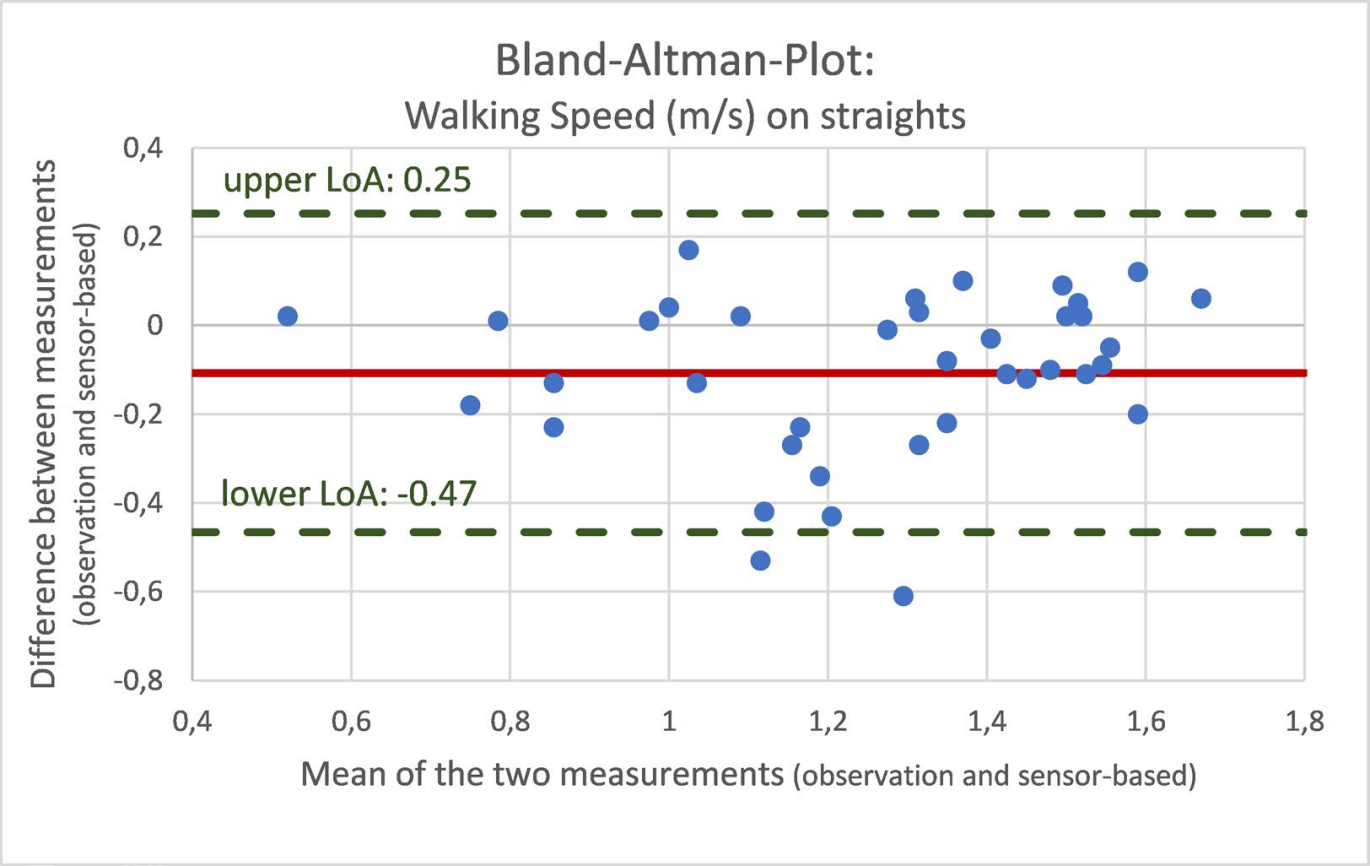
Bland–Altman plot for walking speed on straights comparing the different measurements (video-documented observation and sensor-based assessment)

The Bland–Altman plots illustrate the differences between observed data with video references and sensor-acquired data for total steps (Fig. 2), cadence (Fig. 3) and walking speed (Fig. 4) during straight segments of the 6MWT.

These differences in measures are evenly distributed around the means of the two measurements (steps: 2.7, cadence: − 0.8 steps/min, walking speed: − 0.1 m/s).

### Assessment of fatigability

Regarding the second aim of this study, we assessed fatigability with evaluation of performance over time. Using observed reference data, fatigability was found in 13.2% of all PwMS regarding steps, 2.6% for cadence and 15.8% regarding walking speed (Table 3). Except for one case, all PwMS with detected fatigability are moderately affected patients. One-third of moderately affected PwMS appear to have a fatigability considered decline in walking speed.

**Table 3:** Walking performance over time comparing the first and last minute of the 6MWT using observed values and sensitivity and specificity of the detection of decline by the smartphone sensor

		PwMS total	PwMS mildly affected	PwMS moderately affected
Observed values	Fatigability criterion steps	5/38 (13.2%)	1/23 (4.3%)	4/15 (26.7%)
	Fatigability criterion cadence	1/38 (2.6%)	0/23 (0.0%)	1/15 (6.6%)
	Fatigability criterion walking speed	6/38 (15.8%)	1/23 (4.3%)	5/15 (33.3%)
Sensor data	Sensitivity/specificity steps	0.8/0.97	0/1	1/0.91
	Sensitivity/specificity cadence	1/0.97	1/1	1/0.93
	Sensitivity/specificity walking speed	0.5/1	1/1	0.4/1

In addition, sensitivity and specificity are reported for detection of the 10% decline by the smartphone sensors in Table 3.

### Correlation of sensor-detected 6MWT parameters to fatigue and walking ability

The parameters of the 6MWT and FSMC total, as well as the subscales (motor and cognition) correlated significantly, as demonstrated in Table 4. After multiple comparison correction, the correlation remained significant only for the motor subscale of the FSMC. None of the parameters differs between the first and last minute correlated significantly with the FSMC total or subscores (Table 4).

**Table 4:** Correlations of the FSMC results to sensor-detected 6MWT parameters and change in performance during the 6MWT

		6MWT total steps on straights	6MWT cadence	6MWT walking speed	Difference in steps 1st vs 6th minute	Difference in cadence 1st vs 6th minute	Difference in velocity 1st vs 6th minute
FSMC: Total	Pearson’s correlation	− .397*	− .406*	− .435*	.023	− .103	.026
	*p* value	.014	.012	.006	.892	.537	.875
FSMC: cognitive fatigue	Pearson’s correlation	− .344*	− .352*	− .297	.066	− .135	− .006
	*p* value	.034	.030	.070	.695	.421	.973
FSMC: motor fatigue	Pearson’s correlation	−.500**	− .508^**^	− .501**	.041	− .057	.100
	*p* value	**.001**	**.001**	**.001**	.805	.732	.550

Significant results after multiple comparison corrections in bold. *p < 0.05; **p_cor_ < 0.05

## Discussion

We here are introducing a new smartphone-based approach for gait analysis by validating it in PwMS and healthy controls. We revealed a high validity in patients without gait impairments and controls, whereas gait disability lead to reduced accuracy of the algorithm in PwMS. Furthermore, one-third of the moderately affected PwMS revealed fatigability in the lab-based environment. For the first time, a correlation between objective gait parameters and subjective motor fatigue was demonstrated using acceleration sensors in commonly used smartphones.

### Validity of 6MWT parameters

We assessed the validity of an algorithm developed by the Zentrum für Telemedizin Bad Kissingen (ZTM) processing raw data extracted from mobile sensors. Our data reveal that this approach accurately determines step count and cadence, even among PwMS with moderate levels of impairment. With the MAPE below 3%, these gait parameters can be defined as precise [25]. Apart from the good MAPE rates for steps and cadence, MAPE rates for walking speed were much higher, especially in severely affected PwMS. These results are presumably due to methodological difficulties in assessing velocity with sensor-based technologies, but has to be validated, and hopefully increased, with other methodological approaches or improved accelerators. With a MAPE below 10% in our data, we consider the walking speed detection in mildly affected PwMS and the healthy control group as acceptable.

The Bland–Altman analysis confirmed the agreement of the two methods, with a rather equal distribution of the differences between the individual measurements of the observed and calculated differences.

Therefore, the employed algorithm demonstrates a high accuracy for steps and cadence, even in moderately affected PwMS. Accuracy of walking speed, on the other hand, has to be interpreted with caution, especially for PwMS with evident gait impairment.

### Validity of fatigability criterion

Studies that investigated performance fatigue without wearables compared the walked distance using a distance walked index (DWI) [18, 20] and suggested a decline of -10% from first to sixth minute as a cut-off value for fatigability. We adapted this approach to sensor-based data acquisition, and used the parameters steps, cadence and walking speed for comparison.

Our results show fatigability in up to 16% of the PwMS, which differs between steps, cadence and walking speed, an even in up to 8% of the control participants. Fatigability seems comparable between steps and walking speed, with only one differing participant (*n* = 5/38 for steps, and 6/38 for walking speed), but was only evident in 1/38 PwMS for cadence. This difference was also comparable in the control groups, where no participant was rated as fatigues using cadence decline, but 1/24 or 2/24 for steps and walking speed, respectively.

All but one patient with objective fatigability belong to the moderately affected group, which is comparable to Berg-Hansen et al. [19], that reported similar results using this approach with a decrease only being present in the moderately disabled group, whereas the mild-disability group was stable over time.

Smartphones detected fatigability in moderately affected PwMS using sensor data with high specificity, but varying sensitivity. Most individuals meeting the criterion were identified, although sensitivity was lower in some cases due to the small number of participants showing significant performance decline overall. Notably, sensitivity for walking speed remains too low, especially in moderately affected PwMS, as indicated by the higher MAPE. Individuals with fatigability largely fall into this group. In addition, the assessment suggests that cadence is not an appropriate parameter for examining fatigability.

### Associations to perceived fatigue and fatigability

The majority of PwMS (84.2%) reported fatigue based on the FSMC, which is a much higher rate as compared to the objective assessment of gait-related fatigability. Despite this discrepancy, higher FSMC correlated with lower steps, cadence and velocity in the 6MWT. These results suggest an association between smartphone-detected gait parameters in the 6MWT and subjective perceived fatigue in PwMS. This relation is in accordance with previous studies using other wearables or other measures of fatigue and was expected [21–23], though it had not been demonstrated using smartphone sensors before. Furthermore, as the FSMC is able to differentiate between cognitive and motor fatigue, only motor fatigue maintained significant after adjusting alpha levels according to Bonferroni. These findings underline the validity of the construct of fatigue with a cognitive and physical component, and highlight the potential use of gait analysis in fatigue research.

In contrast to the significant correlation of the total gait scores, we were not able to confirm the decline in performance over time as a potential definition of fatigability, in association to the FSMC. If this is due to the in total small number of PwMS (*n* = 6/38) showing a severe decline in gait compared to the high number of PwMS with pathological fatigue values according to the FSMC (*n* = 32/38) seems speculative, but reasonable. However, this result highlights that a 10% decline of performance is no valid parameter of fatigability in the association to subjective fatigue.

### Limitations

This study has several limitations. The number of patients is quite small, especially with a present gait disturbance. We are aware that this might lead to an underestimation of effects, especially in correlating fatigability in the subgroup with an evident 10% decline in gait performance. Larger samples have to confirm these findings, as well as the usage of gait parameters for fatigability assessment.

In addition, the 6MWT as performed in this study is only a snapshot in a standardized, clinical setting. With having proven the validity of the algorithm in this setting, the goal for futures studies is the assessment in a non-standardized home setting. We are also aware that the groups differ in age and weight. As we only used the healthy controls to validate or approach (aim 1), this does not limit the results in the assessment of fatigue and fatigability in the PwMS group.

Methodologically, we only assessed steps, cadence and walking speed. We are aware that there are many other parameters such as postural stability that are important for gait. As we were interested in the association to fatigue, and due to the limitations of our approach by the used algorithm and by positioning the sensors at a belt, we focused on the commonly used gait parameters. Future research could also investigate if postural stability is related to fatigability.

In addition, step counts at the starting and ending points of analyzed straights could possibly be prone to errors. Future approaches shall assess the entire gait, including curves.

Furthermore, we only used a central position of the smartphone, and further studies investigating other positions in comparison would be interesting. In addition, as only one smartphone type with its built-in sensors was used, this limits a possible generalization for other smartphone models.

## Conclusion

Smartphone-based acceleration data may serve as an objective method for assessing gait fatigability in a lab setting, but its applicability to real-life settings, where cognitive demands might exacerbate fatigue, remains unproven.
